# Antitumor Cell-Complex Vaccines Employing Genetically Modified Tumor Cells and Fibroblasts

**DOI:** 10.3390/toxins6020636

**Published:** 2014-02-19

**Authors:** Antonio Miguel, María José Herrero, Luis Sendra, Rafael Botella, Ana Diaz, Rosa Algás, Salvador F. Aliño

**Affiliations:** 1 Department of Pharmacology, Faculty of Medicine, Universitat de València, Blasco Ibáñez Avenue, 15, Valencia 46010, Spain; E-Mails: matasantonio@hotmail.com (A.M.); luissendra@hotmail.es (L.S.); 2 Instituto Investigación Sanitaria La Fe. Shouth Boulevard, Valencia 46026, Spain; 3 Servicice of Dermatology, Hospital Universitario y Politécnico La Fe. South Boulevard, Valencia 46026, Spain; E-Mail: rbotellaes@gmail.com; 4 Central research unit, Faculty of Medicine, Universitat de València, Blasco Ibáñez Avenue, 15, Valencia 46010, Spain; E-Mail: ana.diaz@uv.es; 5 Servicice of Radiotherapy, Hospital Clínico Universitario, Blasco Ibáñez Avenue, 10, Valencia 46010, Spain; E-Mail: algas_amp@gva.es; 6 Clinical Pharmacology unit, Hospital Universitario y Politécnico La Fe., Valencia 46026, Spain

**Keywords:** cancer vaccines, gene therapy, non-viral, bystander cells, cell complexes

## Abstract

The present study evaluates the immune response mediated by vaccination with cell complexes composed of irradiated B16 tumor cells and mouse fibroblasts genetically modified to produce GM-CSF. The animals were vaccinated with free B16 cells or cell complexes. We employed two gene plasmid constructions: one high producer (pMok) and a low producer (p2F). Tumor transplant was performed by injection of B16 tumor cells. Plasma levels of total IgG and its subtypes were measured by ELISA. Tumor volumes were measured and survival curves were obtained. The study resulted in a cell complex vaccine able to stimulate the immune system to produce specific anti-tumor membrane proteins (TMP) IgG. In the groups vaccinated with cells transfected with the low producer plasmid, IgG production was higher when we used free B16 cell rather than cell complexes. Nonspecific autoimmune response caused by cell complex was not greater than that induced by the tumor cells alone. Groups vaccinated with B16 transfected with low producer plasmid reached a tumor growth delay of 92% (*p* ≤ 0.01). When vaccinated with cell complex, the best group was that transfected with high producer plasmid, reaching a tumor growth inhibition of 56% (*p* ≤ 0.05). Significant survival (40%) was only observed in the groups vaccinated with free transfected B16 cells.

## 1. Introduction

Vaccination with tumor cells genetically modified to produce proinflammatory cytokines has been shown to be effective in several types of cancer [1,2,3,4,5,6,7,8,9,10,11]. Antitumor therapy with modified autologous cells seems in principle to be the best choice. However, it is often very difficult to obtain tumor cells from a patient and cultivate, expand and transfect them correctly in the same patient with guarantees of safety, sterility and adequate production of the transgene. In order to solve this problem, some investigators have proposed transfecting other cells. Such cells are known as bystander cells, which would act as cytokine producer cells [12,13,14,15,16,17,18,19,20,21]. One of the most interesting alternatives is the use of autologous fibroblasts as bystander cells [15,16,17,18,19,20,21], since they can be obtained from patient skin and are easily transfected. We recently developed a vaccine based on cell complexes comprising B16 tumor cells and mouse fibroblasts genetically modified to produce cytokines. To prepare these cell complexes, we employed the cationic polymer PEI (Polyethylenimine, 800 kDa). PEI has been shown to be effective in forming PEI/DNA complexes [21,22,23,24,25,26]. This is possible due to interaction between the negative charges of the DNA and the positive charges of PEI. For this reason PEI is widely used as a vector for gene transfer. The cytoplasmic membrane of the cells also has negative charges that interact with the positive charges of PEI, forming PEI/cell complexes. One advantage of such complexes as anticancer vaccines, in relation to the mix of bystander and tumor cells, is that they facilitate recognition of the tumor cells by the immune system. We have tested these vaccines in a model of mouse melanoma developed in our laboratory, showing the importance of the order of antigen and cytokine presentation [8]. Melanoma is possibly the best candidate for cancer vaccines, because it is a very immunogenic type of malignancy [27]. In this study we employed cells transfected with the GM-CSF gene. This cytokine has been shown to have antitumor activity in preclinical and clinical trials [1,6,28,29,30]. We used two gene plasmid constructions to transfect cells: a high producer (pMok) and a low producer (p2F). The most innovative part of this work is the comparison of both models of vaccination using free B16 tumor cells against Fib-B16/PEI cell complexes that have been genetically modified.

The objective of this study was to demonstrate that vaccines with cell complexes comprising B16 tumor cells and genetically modified fibroblasts are able to generate an antitumor immune response as effective as that produced by vaccines with free genetically modified B16 cells. Our results support that both vaccines mediate a specific antitumor humoral response, but free cell vaccines were more efficient in mediating tumor rejection.

## 2. Results and Discussion

### 2.1. Cytokine Production

**Figure 1:**
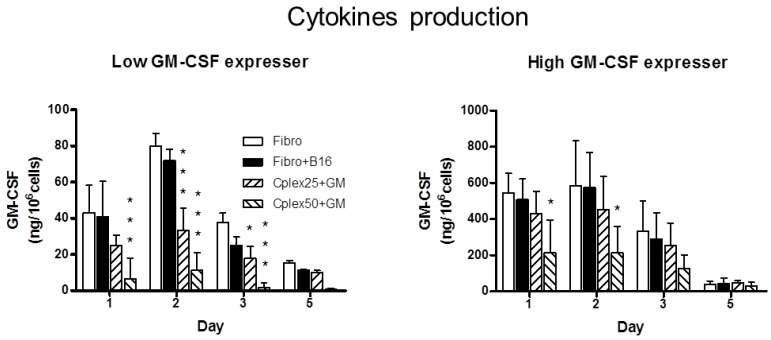
Cytokine production. The figure shows GM-CSF production in fibroblasts transfected with low GM-CSF expresser (**A**), or high GM-CSF expresser plasmid (**B**). GM-CSF production was quantified by ELISA assay of culture medium on days 1, 2, 3 and 5. The production (in ng) at 24 h of GM-CSF/10^6^ cells was measured in the following groups: fibroblasts (Fibro), fibroblasts mixed with B16 cells (Fibro + B16), and cell complex, comprising B16 cells and transfected fibroblasts, using 25 or 50µg/mL Polyethylenimine (PEI) (Cplex25+GM or Cplex50 + GM, respectively). Statistical significance with respect to the Fibro group is indicated: *** *p* < 0.001, and * *p* < 0.05.

Mouse fibroblasts were transfected with two plasmids: a low GM-CSF expresser (p2F *gm-csf*) and a high GM-CSF expresser (pMok *gm-csf*). Cell complexes were formed by B16 tumor cells and transfected fibroblasts employing 25 or 50 µg/mL of PEI. These complexes were incubated during 24 h, and we then collected the culture medium for ELISA assay of GM-CSF. The cytokine production is represented in Figure 1. Figure 1A corresponds to GM-CSF production from fibroblasts transfected with low expresser plasmid (p2F *gm-csf*). The maximum GM-CSF production observed in this figure on the part of the group of cell complexes comprising B16 cells and transfected fibroblasts was 33 ng/10^6^ cells/24 h and 11 ng/10^6^ cells/24 h, employing 25 or 50 µg/mL of PEI, respectively. GM-CSF production from fibroblasts transfected with high GM-CSF expresser plasmid (pMok *gm-csf*) is represented in Figure 1B. In this figure maximum GM-CSF production by the group of cell complexes comprising B16 cells and transfected fibroblasts was 454 ng/10^6^ cells/24 h and 215 ng/10^6^ cells/24 h, employing 25 or 50 µg/mL of PEI, respectively. Fibroblasts treated with 25 µg/mL of PEI maintained cytokine production similar to that of the controls. However, the fibroblasts treated with PEI 50 µg/mL showed a strong decrease in cytokine production compared with the group treated with 25 µg/mL.

### 2.2. Specific Antitumor IgG Production

**Figure 2:**
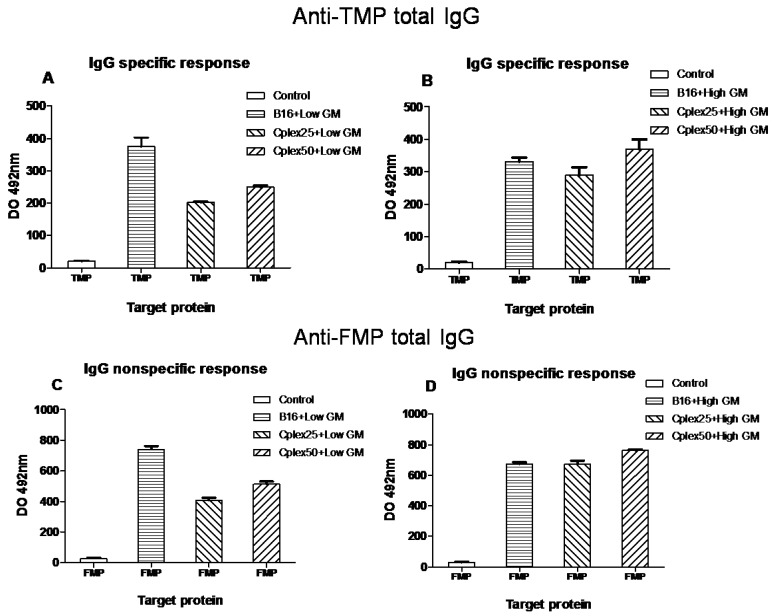
Total specific anti-tumor membrane proteins (TMP) and anti-fibroblast membrane proteins (FMP) IgG production. Mice were vaccinated on days −21, −7 and 7 with respect to tumor implantation (10^5^ B16 wild cells), and blood samples were taken on days −15 and 15, as are described in the experimental section. The groups represented in this figure are mice vaccinated with: control (pool of blood samples, day −15); B16 + Low GM, B16 cells transfected with low GM-CSF expresser plasmid; B16 + High GM, B16 transfected with high expresser plasmid; Cplex25 + Low GM, cell complexes 25 µg/mL PEI transfected with low expresser; Cplex50 + Low GM, cell complexes 50 µg/mL PEI transfected with low expresser; Cplex25 + High GM, cell complexes 25 µg/mL PEI transfected with high expresser; and Cplex50 + High GM, cell complexes 50 µg/mL PEI transfected with high expresser. All groups showed significant differences *versus* the control (*p* < 0.001).

Mice were injected with three vaccine doses on days −21, −14 and 7 with respect to tumor injection (day 0). Blood was collected on days −15 (control) and 15. IgG production was measured in these blood samples. Specific IgG production among antitumor membrane proteins (TMP) and anti-fibroblast membrane proteins (FMP) is shown in Figure 2. The vaccine groups are defined in the figure. Total IgG production was always higher on day 15 after tumor injection than on day−15 (control). As expected, in all cases IgG production was higher in the vaccinated groups than in the control group. Figure 2A,C shows total IgG production in animals vaccinated with B16 cells or cell complexes transfected with low GM-CSF producer plasmid. In these figures, maximum IgG production was reached by the group vaccinated with transfected B16 cells only. IgG production in the groups vaccinated with cell complexes employing 25 or 50 µg/mL of PEI was very similar. Figure 2B,D represents IgG production in animals vaccinated with B16 cells or cell complexes transfected with high expresser plasmid. In this case, all vaccinated groups produced the same quantity of IgG. 

In all groups, including free transfected B16 cells, the production of specific anti-FMP IgG was higher than the production of specific anti-TMP IgG.

**Figure 3:**
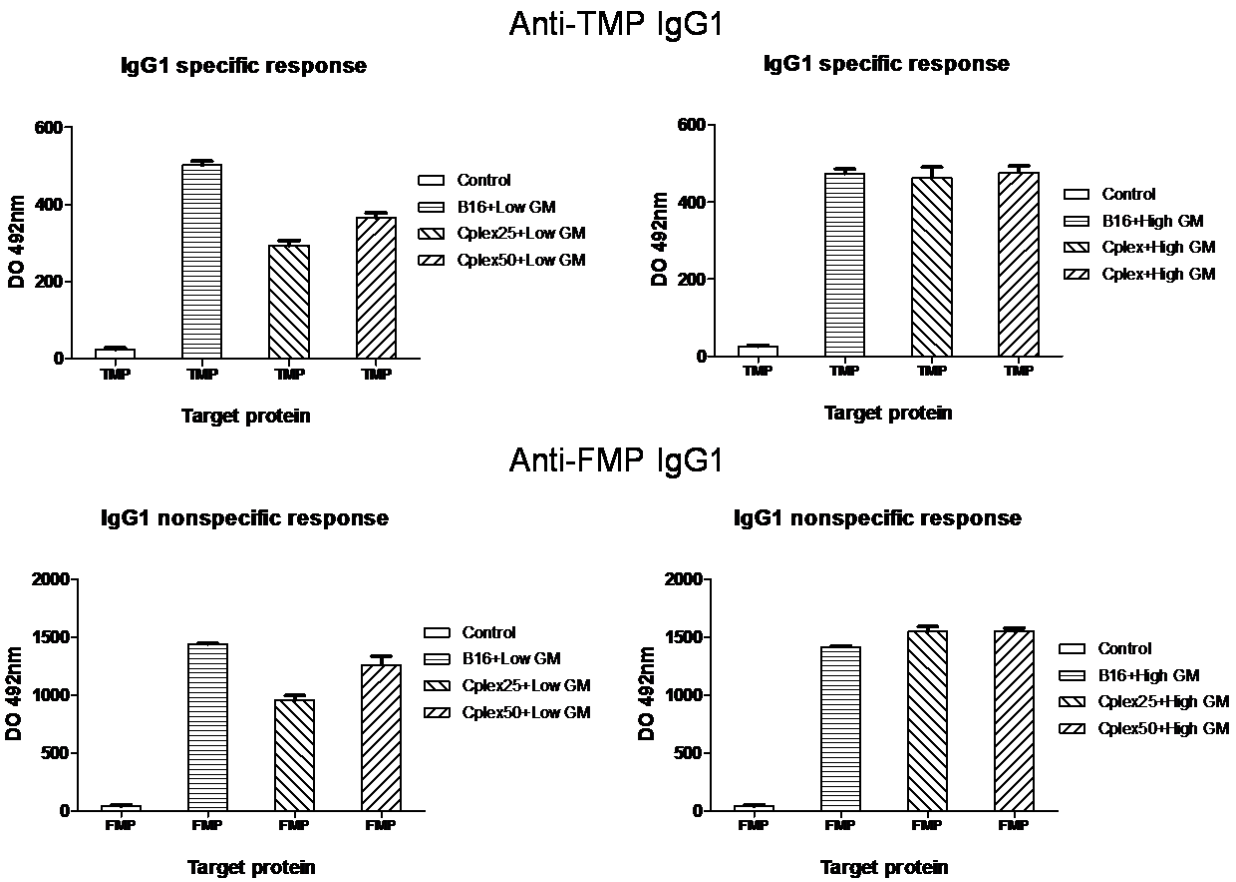
Specific anti-TMP and anti-FMP IgG1 production. IgG1 among anti-TMP (**A** and **B**) and anti-FMP (**C** and **D**) from vaccinated mice are represented in this figure. The treatment groups are described in Figure 2. All groups showed significant differences *versus* the control (*p* < 0.001).

**Figure 4:**
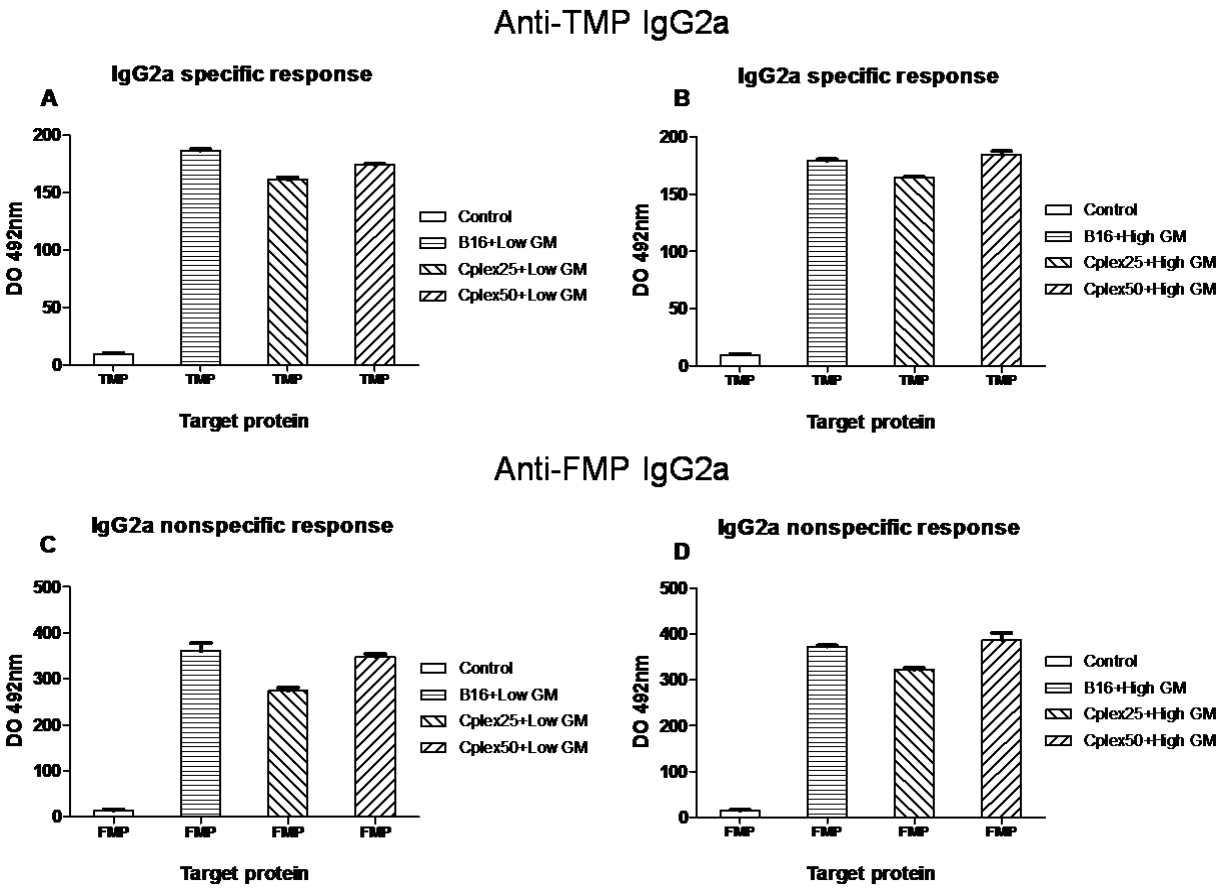
Specific anti-TMP and anti-FMP IgG2a production. IgG2a among anti-TMP (**A** and **B**) and anti-FMP (**C** and **D**) from vaccinated mice are represented in this figure. The treatment groups are described in Figure 2. All groups showed significant differences *versus* the control (*p* < 0.001).

IgG subtypes production is represented in Figure 3 and Figure 4. Figure 3 shows IgG1 production and Figure 4 represents IgG2a production. The results shown in both figures are very similar, though IgG2a production is lower than IgG1 production.

The differences in IgG1 and IgG2a production between all groups of animals are similar to those obtained with total IgG production.

In all cases, IgG production in groups vaccinated with cell complexes was never higher than IgG production in groups vaccinated with free B16 cells. Units of DO 492 are relative; for this reason, the units are not directly comparable between figures.

### 2.3. Tumor Volume

**Figure 5:**
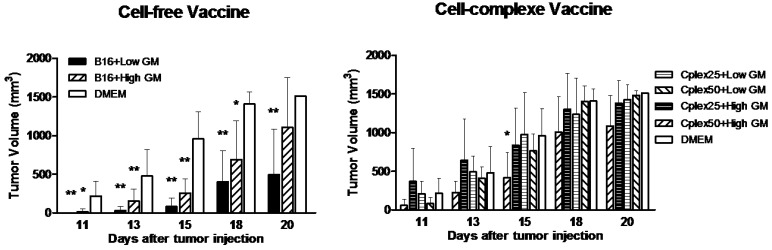
Tumor volume in free cell vaccine and cell complex vaccine. To measure tumor growth, tumor volume was calculated as described in the Experimental Section. Vaccine groups are defined in Figure 2 but in this case the control group was mice injected with DMEM. Statistical significance with respect to the DMEM group is indicated: ** *p* < 0.01, and * *p* < 0.05.

After tumor injection, tumor growth was monitored visually, and tumor volume was measured in all animals with a caliper. The tumor started to be visible from day 11 after tumor implantation. Figure 5A shows the results of tumor volume in groups vaccinated with free B16 cells. The best results were obtained with the group vaccinated with B16 cells transfected with low GM-CSF expresser plasmid, reaching 92%, 71% and 67% tumor growth inhibition *versus* the control group (DMEM) on days 15, 18 and 20, respectively (*p* ≤ 0.01). The group vaccinated with B16 cells transfected with high GM-CSF producer plasmid reached 70% and 51% tumor growth delay *versus* the control group on days 15 (*p* ≤ 0.01) and 18 (*p* ≤ 0.05). Figure 5B shows the results of tumor volume in groups vaccinated with cell complexes. The group vaccinated with cell complexes comprising B16 cells and fibroblasts transfected with high GM-CSF expresser plasmid and PEI 50 µg/mL reached 56% (*p* ≤ 0.05), 28% and 28% tumor growth inhibition *versus* the control group on days 15, 18 and 20, respectively. The others groups vaccinated with cell complexes did not delay growth of the tumor.

### 2.4. Animal Survival

**Figure 6:**
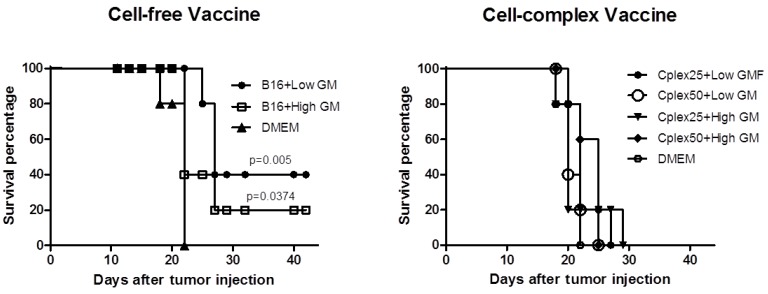
Animal survival with free cell vaccine and cell complex vaccine. The plot shows survival of the groups described in Figure 2.

Survival curves from groups vaccinated with free B16 cells are shown in Figure 6A. The group vaccinated with B16 cells transfected with low GM-CSF expresser plasmid reached 40% overall survival (*p* < 0.01), while the group with B16 cells transfected with high GM-CSF producer plasmid reached 20% overall survival (*p* < 0.05). The curves were consistent with the inhibition of tumor growth. Figure 6B represents animal survival in groups vaccinated with cell complexes. With cell complex vaccines none of the animals reached overall survival, but the results in the vaccinated groups were always better than those in the control group.

In this study we evaluated the antitumor response in mice vaccinated with cell complexes comprising B16 tumor cells and genetically modified mouse fibroblasts. These complexes offer advantages when low availability of patient tumor cells exists.

In this work we compared the antitumor response produced by this cell complex vaccine with a vaccine based on free B16 cells and modified with two plasmids (low and high GM-CSF expressers). We observed that the following: (a) the humoral antitumor response was similar in groups vaccinated with B16 cells alone or with cell complexes; (b) the nonspecific immune response was very similar in both groups; (c) both vaccines delayed tumor growth, but vaccination with free cells mediated better antitumor response; and (d) only free cell vaccines achieved survival success.

The fibroblasts forming complexes were transfected with two plasmids: a low GM-CSF expresser (p2F *gm-csf*) and a high GM-CSF expresser (pMok *gm-csf*). The gene promoter of pMok is more potent, allowing greater gene expression, whereas p2F offers the possibility of transporting two genes as an advantage. We showed that these complexes are able to produce GM-CSF at levels that have been shown to have antitumor activity (GM-CSF > 35 ng /10^6^ cells/24 h) [28,31]. We confirmed that cytokine production is 10 times higher in cells transfected with the high expresser plasmid than with the low expresser. 

In order to evaluate the production of specific anti-TMP (tumor membrane proteins) IgG and its subtypes in mice vaccinated with both genetically modified free and cell complex vaccines, we collected blood samples from days −15 and 15. The production of total specific anti-TMP IgG and its subtypes was higher in all groups vaccinated with cell complexes than in the control group. This fact demonstrated that cell complexes are capable of producing a humoral anti-tumor response. In addition, the production of specific anti-FMP (fibroblast membrane proteins) IgG in groups vaccinated with free B16 cells indicates that vaccination with tumor cells generates across immune response between B16 and the fibroblasts. This is due to the fact that the tumor cell shares many epitopes with healthy cells of mice. On the other hand, in groups vaccinated with B16 cells transfected with low GM-CSF expresser plasmid, the generated humoral antitumor response was greater than the response generated by groups vaccinated with cell complexes transfected with the same plasmid. We consider that this result is due to the decrease in the production of GM-CSF caused by the presence of PEI in the complexes, since we observed that increased PEI mediates reductions in the production of this cytokine. We have also observed the reduction of IL12 cytokine and we think that this could be due to PEI toxicity. In this respect, however, IgG production for both vaccines transfected with high GM-CSF expresser was very similar. Probably this effect is due to the high GM-CSF production in this case, avoiding the negative effect of PEI.

When animals were vaccinated with free B16 tumor cells, the best results were obtained with the group transfected with the low GM-CSF expresser plasmid; reaching a significant 92% of tumor growth delay on day 20. This result reinforces the idea that high doses of GM-CSF reached in free cells transfected with high expresser plasmid must be mediating negative effects [6,31]. Accordingly, in the groups vaccinated with cell complexes in which PEI reduces GM-CSF production, the best results were obtained using high GM-CSF expresser cells (56% tumor growth delay). 

The survival curves are consistent with the results of tumor development. Only the groups vaccinated with free B16 cells transfected with low expresser plasmid and high expresser achieved 40% and 20% overall survival, respectively. The failure of the antitumor response mediated by cell complexes could be due to low GM-CSF expression and by the natural production of immune suppressive cytokines such as TGF beta by fibroblasts.

The use of these cell complexes as cancer vaccine could be possible because the cell complexes do not increase the cross-immune response observed with tumor cells alone, and are able to generate specific antitumor immune response. Although our results suggest that cell complexes mediate a better humoral antitumor immune response than cell mediated tumor rejection, additional studies with different doses of B16 forming complexes could help to achieve the equal antitumor efficacy achieved with genetically modified free tumor cells. These cell complexes could have other applications, for example as adjuvants in vaccines or as sustained-release proteins systems.

## 3. Experimental Section

### 3.1. Plasmids

The p2F plasmid employed was derived from the pVITRO2 base plasmid (Invivogen, Toulouse, France), with the *gm-csf* gene inserted. pVITRO2 allows the co-expression of two genes and contains two human ferritin composite promoters, FerH (heavy chain) and FerL (light chain), combined to the SV40 and CMV enhancers, respectively, and the resistance to hygromycin gene. Another plasmid was also employed, pMok *gm-csf* (Mologen, Germany), in order to obtain another construct expressing *mGM-CSF,* that had previously been successfully used in our laboratory. This pMok *gm-csf* plasmid contains the kanamycin resistance gene and the murine *gm-csf* gene, controlled by the CMV promoter. pMok produces 10 times more GM-CSF with respect to p2F.

### 3.2. Cells and Transfection Procedure

B16 murine melanoma cells were mainly used for this study. These cells are syngeneic with the animals used for vaccination, *i.e.*, C57BL/6 mice (Harlan, Gannat, France). The mouse fibroblasts were obtained by our laboratory from explants obtained from skin of the C57BL/6 mouse. In brief, small biopsies of the epidermis of the mouse were placed on the bottom of 6-well culture plates. Once the biopsy was adhered, medium was carefully added. The biopsy was removed from culture when the fibroblasts were released from tissue in the next weeks.

The cells were transfected by means of a chemical procedure based on PEI 25 kDa (polyethyleneimine, Sigma, Madrid, Spain) polyplexes (DNA:PEI, 1:1.41) with 5 μg/mL of plasmids, as previously described [25,32]. The transfection percentage with this method lies between 20% and 40% of total cells, as observed using the reporter *EGFP* gene (data not shown). Cells were transfected when more than 80% confluence was reached in their flasks. 

### 3.3. Cytokine Expression ELISA Assay

GM-CSF production from the transfected fibroblasts or transfected B16 cells was determined by enzyme linked immunosorbent assay (ELISA), performed on supernatant samples of the culture media taken 24 h post-transfection and prior to cell detachment and irradiation. The BD OptEIA ELISA kit for mGM-CSF (Pharmingen, BD Biosciences, Madrid, Spain) was used. A time point of 5 days was chosen on the basis of prior experimental results, assessed to study cytokine production over time, using the referred transfection conditions [25,32], in order to guarantee adequate GM-CSF production according to the literature, *i.e.*, >35 ng/10^6^ cells/24 h [28,31].

### 3.4. Specific Anti-TMP and Anti-FMP IgG ELISA Assay

Measurement of IgG and IgG1 and IgG2a subtype antibodies against TMP (Tumor Membrane Proteins) or FMP (Fibroblast Membrane Proteins) was performed in serum samples by specific ELISA, as previously described [8,29]. TMP and FMP are an extract of the hydrophilic membrane proteins of the irradiated B16 cells and fibroblasts, respectively. Thus, with these ELISA assays we tested the specific and nonspecific immune responses to our vaccine treatment. All samples were assayed in duplicate, allowing estimation of the mean Optical Density value and standard deviation.

### 3.5. Cell Complexes Formation

Cell complexes were formed employing B16 tumor cells, fibroblasts and PEI (polyethylenimine 800 kDa, Sigma, Spain). Tumor cells (2 × 10^6^ cells/mL) were incubated in the presence of PEI (25 or 50 µg/mL) and incubated during 15 min. The fibroblasts (10^6^ cells/mL) were added and incubated another 15 min. 

### 3.6. Vaccination Design

C57BL/6 mice (8–10 weeks old) kept under standard laboratory conditions were housed 5 mice per cage. The experimental project was approved by the Biological Research Committee of the University of Valencia (Valencia, Spain). In all cases, mice were vaccinated subcutaneously (right leg) with a single dose per week, in weeks −3, −1 and +1 (days −21, −7 and +7), with respect to tumor injection (day 0) with 10^5^ wild type B16 cells in the left leg. The number of cells employed in each vaccine dose was 2 × 10^5^ B16 cells per mouse or cell complexes comprising 2 × 10^5^ B16 cells and 2 × 10^5^ fibroblasts in 200 μL DMEM.

In all vaccination experiments, blood samples were taken from all the animals and pooled for the same group at each time point. The samples were taken on days −15 (before tumor implantation, serving as base level or control in each group) and +15 (one week after the third and last dose was administered). Plasma was obtained by centrifugation at 3000 rpm for 5 min., and kept at −20 °C until use.

The treatment groups were free cells or cell complexes transfected with low GM-CSF expresser plasmid (p2F *gm-csf*) or high GM-CSF expresser plasmid (pMok *gm-csf*). As control we employed 200 µL DMEM.

### 3.7. Tumor Growth Measurement and Survival

Tumor growth in mice was monitored visually and measured with a caliper in two dimensions: A (long diameter) and B (short diameter). Tumor volume was calculated with the formula: V = (A × B^2^)/2, and expressed in mm^3^. Animals were collected at date of death to construct the survival curves. Mice were sacrificed when the tumor volume reached 1.5 cm^3^.

### 3.8. Statistical Analysis

Statistical comparison of the tumor growth inhibition results between different groups with respect to control was based on a nonparametric analysis with the Mann-Whitney U-test (95% confidence interval, 95%CI). The same test was applied to the results of the IgG ELISA assays. Statistical comparison of the GM-CSF production results in the different treatment groups was based on two-tailed analysis of variance (ANOVA) with Bonferroni *post hoc* testing (95%CI). 

Significance in relation to survival was analyzed using the Kaplan-Meier survival curves and log-rank nonparametric test.

All the tests and plots were performed with the Graph Pad Prism 4^®^ package.

## 4. Conclusions

- Cell complexes comprising B16 tumor cells and mouse fibroblasts, genetically modified, produce sustained GM-CSF amounts. 

- Cell complexes producing GM-CSF are able to induce an antitumor immune response.

- In mice vaccinated with cell GM-CSF producer complexes the nonspecific humoral immune response is not higher than with B16 free cells.

- Vaccination with cell complexes is more effective using GM-CSF high expresser plasmid than low expresser plasmid.

- The vaccine doses of GM-CSF producer cell complexes tested in this study delay tumor growth, but are not sufficient to avoid the tumor development and achieve animal survival.
